# Transcriptional analysis and target genes discovery of *Pseudomonas aeruginosa* biofilm developed ex vivo chronic wound model

**DOI:** 10.1186/s13568-021-01317-2

**Published:** 2021-11-27

**Authors:** Xiaojuan Tan, Xi Cheng, Mei Hu, Yifan Zhang, Aiqun Jia, Jinwei Zhou, Guoping Zhu

**Affiliations:** 1 Anhui Provincial Key Laboratory of Molecular Enzymology and Mechanism of Major Diseases, Anhui Normal University, Wuhu, 241000 Anhui China; 2 Key Laboratory of Biomedicine in Gene Diseases and Health of Anhui Higher Education Institutes, College of Life Sciences, Anhui Normal University, Wuhu, 241000 Anhui China; 3 School of Life and Pharmaceutical Sciences, State Key Laboratory of Marine Resource Utilization in South China Sea, Hainan University, Haikou, 570228 China; 4 School of Food and Biology Engineering, Xuzhou University of Technology, Xuzhou, 221018 China

**Keywords:** *Pseudomonas aeruginosa*, Biofilms, Chronic wound infections, Alkaline phosphatase, Nitric oxide

## Abstract

Bacterial biofilms formation is one of the major reasons for treatment failure in chronic wound infections. Therefore, diagnostic biomarkers remain the best option for prevention and treatment of chronic wound infections by biofilms. Herein, *Pseudomonas aeruginosa* PAO1 was used to mimic biofilm development in porcine skin explants wells as ex vivo wound model. The microscopic imaging showed that PAO1 in porcine skin explants wells formed micro-colonies at 24 h, developed mushroom-like structure at 48 h, and at 72 h mushroom-like structure disappeared, remaining a thin bacterial lawn. RNA-seq data analysis revealed that the expression levels of genes involved in the type II *hxc* secretion system were significantly higher in biofilms than in planktonic cells, especially the expression of *lap*A encoding alkaline phosphatase. However, the expression levels of genes associated with denitrification pathway were markedly decreased in biofilms, especially the transcription of *nir*S encoding nitrite reductase to produce nitric oxide (NO). Therefore, their expressions and products were further detected using RT-qPCR and biochemical assays, respectively. The results found that the expression of *lap*A and alkaline phosphatase activity were induced, but the expression of *nir*S and intracellular NO were reduced at the whole biofilms cycle. The study indicates that LapA and NO would play an important role for *P. aeruginosa* biofilm formation in chronic wound infections. LapA would serve as potential target to monitor chronic wound infections by *P. aeruginosa* biofilms. Inducing NO would be used to treat chronic wound infections due to *P. aeruginosa* biofilms.

**Supplementary Information:**

The online version contains supplementary material available at 10.1186/s13568-021-01317-2.

## Introduction

As the population ages, chronic wounds have become a major worldwide healthcare burden (Wu et al. 2019). Major types of chronic wound, including diabetic ulcers, pressure injuries, and venous stasis ulcers, are difficult to control either by immunological response or by antimicrobial therapy (Milho et al. 2019). Among the reasons for treatment failure in chronic wound infections, bacterial biofilms formation is one of the major reasons (Bjarnsholt 2013). Previous research revealed that most chronic wounds (greater than 60%) have been associated with biofilms, while only 6% of acute wounds were characterized as biofilm containing (James et al. 2008).

*Pseudomonas aeruginosa* is one of the predominant bacteria in chronic wounds regardless of the etiology of the wound or type of isolated sampling (Ruffin and Brochiero 2019). In addition, this pathogen has been listed by World Health Organization as a priority critical pathogen (Ball et al. 2002). Previous researches showed that *P. aeruginosa* can easily develop biofilms on abiotic and biotic surfaces Parsek and Greenberg 1999; Bagge et al. 2004; Zhou et al. 2021). Although the molecular mechanisms of *P. aeruginosa* biofilms development on the abiotic surfaces have been identified, the molecular mechanisms underlying chronic wound infections in biofilms by *P. aeruginosa* are still unclear. Importantly, more and more evidences suggest that *in vitro* biofilm models using non-biological substrate have the disadvantage that the data generated may not accurately reflect the real mechanism of biofilm formation in chronic wounds and efficacy of antimicrobial agents on biofilms in wounds. Therefore, a better understanding of the biofilm formation mechanism of chronic wound-associated *P. aeruginosa* is important to understand the high persistence and resistance of biofilm cells using *ex vivo* porcine skin explants model instead of in vitro models (Yang et al. 2013).

In this work, the specific transcriptomic responses of *P. aeruginosa* biofilm cells to ex vivo wound model with porcine skin explants were evaluated by RNA sequencing. And then, the results obtained were verified with RT-qPCR and biochemical experiments, respectively. This study allowed us to decipher the differentially expressed genes and potential mechanisms developed by biofilms to adapt to the different physiological environments. Our data represent a global view of transcriptomic regulation in *P. aeruginosa* biofilms response to chronic wound infections. Considering finding adequate and more effective therapeutic approaches to treat *P. aeruginosa* biofilm infection, further understanding of the molecular mechanisms underlying biofilm formation and then discovering the target genes could lead to the development of novel treatment strategies to control the dissemination of this pathogen.

## Materials and methods

### Bacterial strain

*Pseudomonas aeruginosa* PAO1 was used as a model strain in this work to study biofilm developed in chronic wounds. *Pseudomonas* isolation agar (PIA) was used to culture the colonies in biofilms. LB medium was used for routine culture and growth.

### The construction of ex vivo wound model with porcine skin explants

*Ex vivo* wound model with the porcine skin explants was established as previously described with some modifications (Yang et al. 2013; Wang et al. 2018). Briefly, frozen skin, obtained from local supermarket, was used as wound model substrate. The porcine skin explants (10 mm) containing central 4 mm diameter × 1 mm deep wells were prepared using a disposable skin biopsy punch. And then, the explants were immediately washed with 200 mL sterile water for three times. After which, each set of six explants was placed into sterile beakers containing 100 mL of 4% sodium hypochlorite solution and incubated at room temperature for 40 min at first time and 20 min for second. After treatment with disinfectant solution, each set of six explants was washed with 50 mL sterile water for six times. All of explants were exposed to UV light in the biosafety hood for around 30 min to remove excess water on the surface. Each set of three explants was transferred to soft agar plates (only containing 0.5% agar) with 25 µg/mL irgasan in order to inhibit the skin native strains growth.

### Biofilm development and quantification

**Fig. 1:**
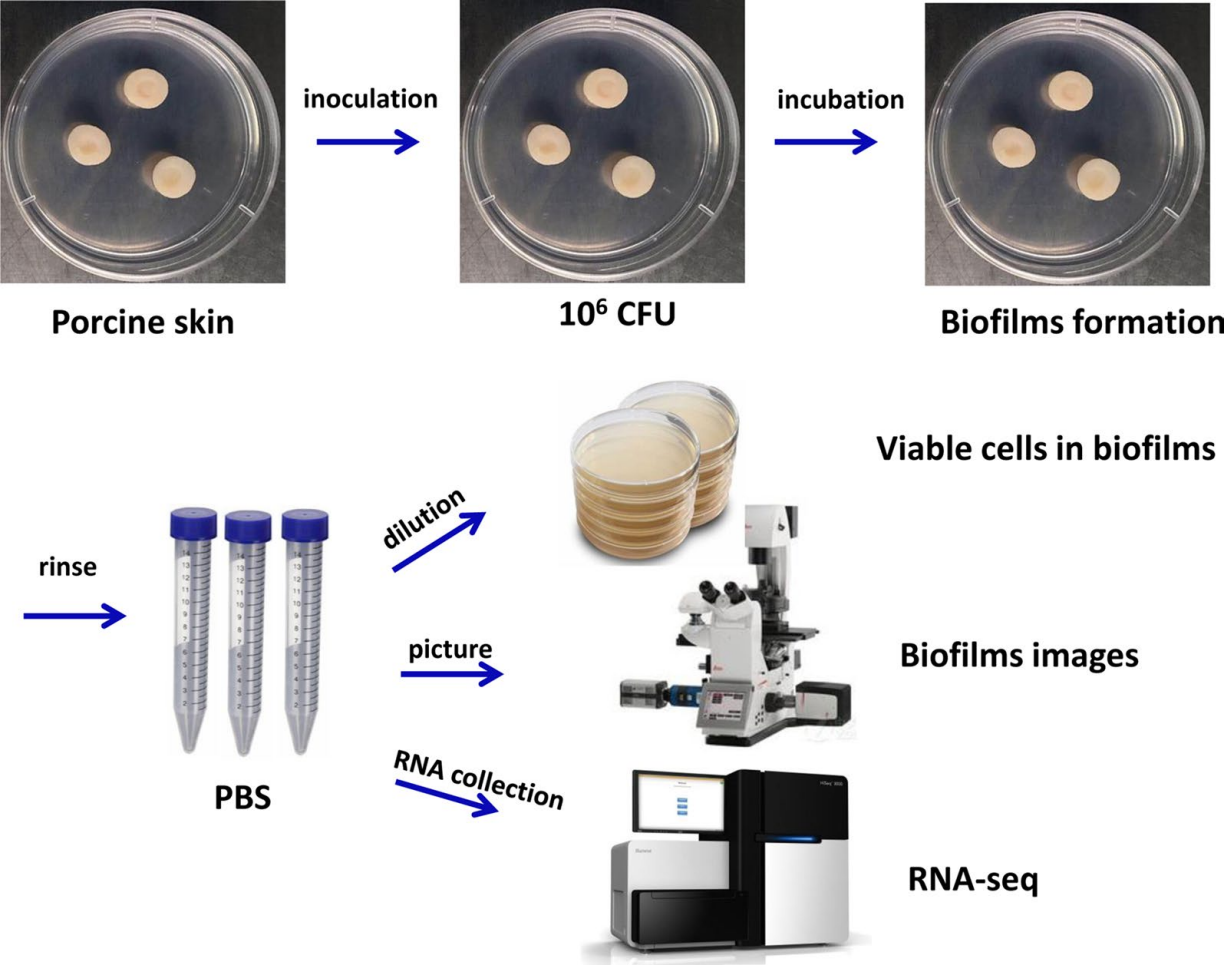
The ex vivo biofilms model to assess *Pseudomonas aeruginosa* biofilm developed in chronic wounds

This assay was performed as previously described with a few modifications (Yang et al. 2013) (Fig. 1). Briefly, 10 µL (about 10^6^ CFU) of log-phase PAO1 culture was added to each explant well. The soft agar plates (only containing 0.5% agar) were statically incubated at 37 ^o^C for 24 h, 48 h, and 72 h, respectively. All of the explants were transferred to fresh soft agar plates with 25 µg/mL irgasan each day. 10 µL of LB medium was added to explant well and incubated at the same conditions as negative control. For quantification of biofilm developed in the porcine skin explant wells, the explants were gently rinsed with 10 mL sterile PBS for three times to remove loosely adhered cells. After which, the explants were sonicated in 2 mL tubes containing 1 mL sterile PBS for 30 s and then mixed vigorously for 30 s. Proper dilutions were prepared with sterile PBS and plated on PIA plates. The plates were incubated at 37 ^o^C overnight and then bacterial counting was performed. In addition, one set of rinsed explants was stained with SYTO9 nucleic acid stain (Life technologies, USA) according to manufacturer’s recommended protocol. Imaging of biofilms in explant well was performed with fluorescence microscope (Leica Microsystem, Germany). The locations were chosen at the center of reservoir to avoid edge effects.

### RNA extraction and sequencing

Six samples (3 for planktonic and 3 for biofilm) were used for RNA sequencing. For planktonic samples, all samples used for RNA sequencing were prepared in test tubes. 2 mL of diluted overnight culture (1:100 dilution) with LB medium in 15 mL tube was incubated at 37 ^o^C for 3.5 h with shaking at 150 rpm until log phase. The cell suspensions were centrifuged at 8000 rpm for 5 min and the supernatants were discarded. The pellets were stored at − 80 ^o^C until they were required. For biofilm samples, according to the results of PAO1 biofilms developed in porcine skin explant wells, the mature biofilm samples cultured for 48 h were collected for transcriptome study. The collecting method was as follows: the explants were gently rinsed with 10 mL sterile PBS for three times, sonicated in 2 mL tubes containing 1 mL sterile PBS for 30 s and mixed vigorously for 30 s. The pellets were washed with sterile PBS for three times to remove fat from explants. Finally, the pellets from six explants wells were merged into one sample. Three samples were stored at − 80 ^o^C until they were required.

Total RNA was extracted using TRIzol® Reagent (Invitrogen, USA) according to the manufacturer’s instructions and genomic DNA was removed using DNase I (TaKara, China). Finally, each total RNA sample was suspended in 30 µL RNase-free water and their qualities were determined using 2100 Bioanalyser (Agilent, USA) and the ND-2000 (NanoDrop Technologies). Only high-quality RNA (OD260/280 = 1.8 ~ 2.0, OD260/230 ≥ 2.0, RIN ≥ 6.5, 28 S:18 S ≥ 1.0, ≥ 100 ng/µL, ≥ 2 µg) was used to construct sequencing library in Shanghai Majorbio Bio-pharm Technology Co., Ltd.

RNA-seq library was prepared following TruSeq^TM^ RNA sample preparation Kit from Illumina (San Diego, CA, USA) using 2 µg of total RNA. Briefly, ribosomal RNA (rRNA) depletion is performed by Ribo-Zero Magnetic kit (epicenter) and then all mRNAs were broken into short fragments (200 nt) by adding fragmentation buffer firstly. Secondly double-stranded cDNA was synthesized using a SuperScript double-stranded cDNA synthesis kit (Invitrogen, CA, USA) with random hexamer primers (Illumina, CA, USA). The synthesized cDNA was then subjected to end-repair, phosphorylation and ‘A’ base addition according to Illumina’s library construction protocol. Libraries were selected for cDNA target fragments of 200 bp on 2% Low Range Ultra Agarose followed by PCR amplified using Phusion DNA polymerase (NEB, England) for 15 PCR cycles. After quantified by TBS380, paired-end RNA-seq sequencing library was sequenced with the Illumina HiSeq×TEN (2 × 150 bp read length). The processing of original images to sequences, base-calling, and quality value calculations were performed using the Illumina GA Pipeline (version 1.6), in which 150 bp paired-end reads were obtained. A Perl program was written to select clean reads by removing low-quality sequences, reads with more than 5% of N bases (unknown bases), and reads containing adaptor sequences.

### RNA-Seq data analysis

The data generated from Illumina platform were used for bioinformatics analysis. All of the analyses were performed using the free online platform of Majorbio Cloud Platform (www.majorbio.com) from Shanghai Majorbio Bio-pharm Technology Co.,Ltd. Briefly, high quality reads in each sample were aligned to *P. aeruginosa* PAO1 genome (GenBank accession number NC_002516.2) with Bowti2 tool (Langmead and Salzberg 2012). For rRNA contamination assessment, randomly selected 10,000 raw reads in each sample are aligned to Rfam database with blast method. Based on the annotation results, percentage of rRNA in each sample is calculated. In addition, gene expression levels were calculated using RSEM (Li and Dewey 2011). Differential expression analysis was carried out using the statistical software R package DESeq2 (Love et al. 2014) with following parameters: (1) Benjamini-Hochberg (BH) adjusted *P*_value (padj) must be less than 10^−3^; (2) a log_2_FC between biofilm and planktonic cells is greater than two-fold; and (3) the normalized count value of each gene must be more than 1.00 in either of the conditions.

### Biological interactions

In order to determine the function of differentially expressed genes, GO analysis and KEGG pathway enrichment were performed using STRING (version 10.0) (Szklarczyk et al. 2015). Classes with *P* ≤ 0.05, FDR-adjusted, were considered statistically significant for enrichment. Gene clusters were carried out using STRING with confidence score lager than 0.90 for differentially expressed genes, and the results obtained were observed using Cytoscape (version 3.8.0) with MCODE plugin (Cline et al. 2007).

### RT-qPCR assay

To validate RNA-seq data, RT-qPCR was performed to quantify the transcription of 11 differentially expressed genes by new total RNA samples obtained from independent experiments performed under the same biological conditions. Oligonucleotide primers were designed using Primer 3 and the sequences are shown in Additional file 1: Table S7. The RT-qPCR was carried out in a 20 µL system using MonAmpTM SYBR Green qPCR mix (Monad, China) as recommended by the manufacturer. These reactions were performed using LightCycler 96 Instrument (Roche Diagnostics, USA) with the following cycle parameters: 95 °C for 30 s, followed by 40 cycles of 95 °C for 5 s, 60 °C for 30 s, and 95 °C for 15 s. All measurements were performed in triplicate and the housekeeping gene *rec*A was used as internal reference for normalization. Fold change between biofilm and planktonic samples was calculated using −∆∆Ct method.

### Alkaline phosphatase detection assay

To measure alkaline phosphatase secreted by biofilms and planktonic cells, the supernatants were collected and then were detected with Alkaline Phosphatase Assay Kit (Beyotime, China) as recommended by the manufacturer. The amount of alkaline phosphatase was defined as the µmoles of *p*-nitrophenol liberated from *p*-nitrophenyl phosphate at the specific time and the data was normalized to log_10_CFU.

### Intracellular NO detection assay

To detect intracellular NO level in biofilms and planktonic cells, the pellet samples were collected and then were treated with NO probe DAF-FM DA (Beyotime, China) for 1 h according to the manufacturer’s instructions. After washing biofilms with sterile PBS, the intracellular NO was measured with fluorescence microplate reader (excision: 480 nm; emission: 515 nm). The samples without treated by NO probe were used as negative controls. The obtained values were normalized to log_10_CFU.

### Statistical analysis

All assays were carried out at least three times with independently unless otherwise stated. The results obtained were summarized in figures and tables as mean ± standard deviations. Statistical significance was evaluated using a two-tailed Student’s *t* test. A *P*-value < 0.05 was considered significant.

## Results

### The construction of porcine skin explant wells wound model for biofilm development

**Fig. 2:**
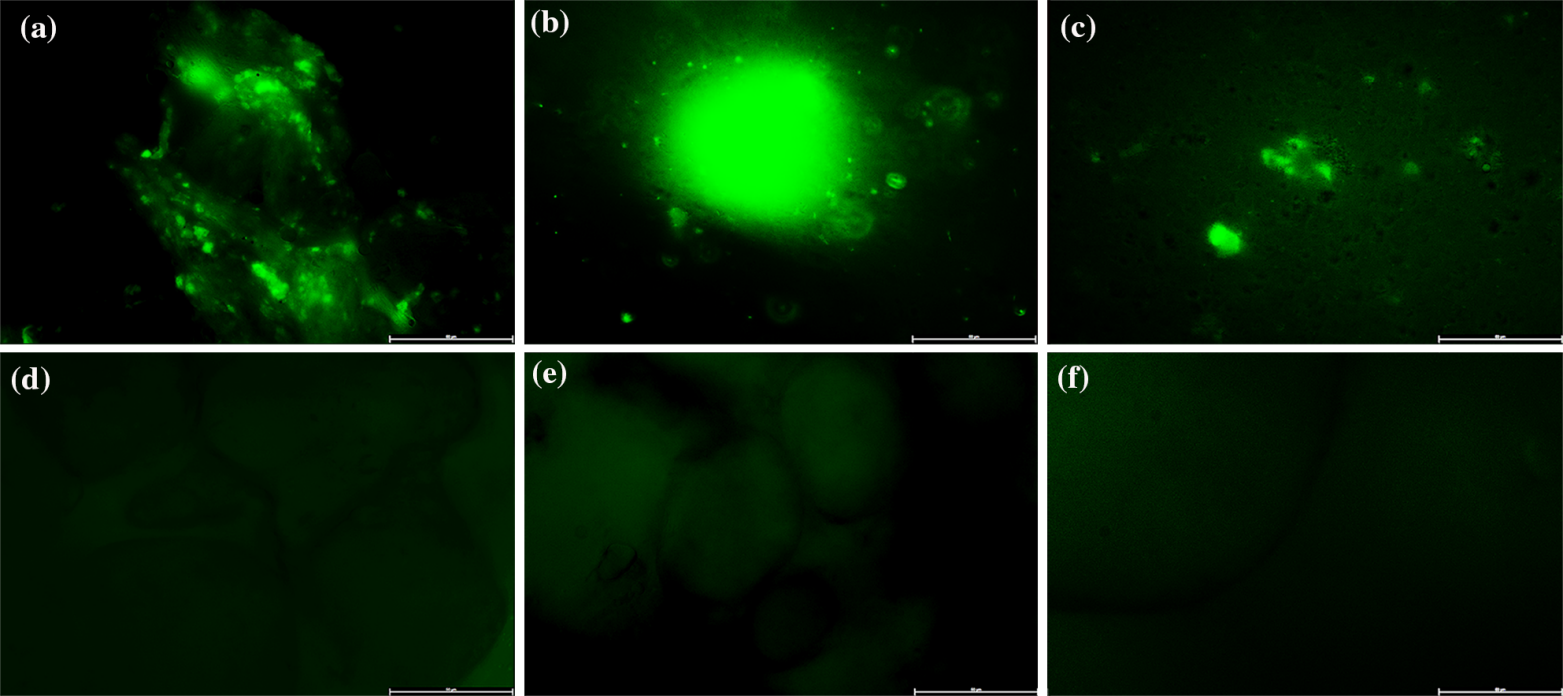
Fluorescence images of *P. aeruginosa* PAO1 biofilm developed in porcine skin explant wells. **a**–**c** Porcine skin explants incubated with PAO1 for 24 h, 48 h, and 72 h, respectively; **d**–**f** porcine skin explants incubated with LB medium as negative controls for 24 h, 48 h, and 72 h, respectively. Scale bars are indicated in 50 µm

The ex vivo biofilm formation by *P. aeruginosa* PAO1 in porcine skin explant wells was monitored using fluorescent microscopic imaging and plating. The fluorescent imaging showed that the PAO1 strain formed micro-colonies for 24 h in explant wells, developed mushroom-like structure at 48 h, and at 72 h mushroom-like structure disappeared, remaining a thin bacterial lawn (Fig. 2). The results of plating determined that when incubating for 48 h, the count of bacterial in explant wells is the most than incubating 24 and 72 h, which is consistent with the results of imaging (Additional file 1: Fig. S1). Under the tested conditions, it was found that within 72 h, the PAO1 went through the whole biofilm developing cycle of “initial attachment, micro-colonies, mature biofilm, and dispersal” (Mihai et al. 2019). To assess potential contamination from other microorganisms, the porcine skin explant wells incubated with LB medium were imaged and plated after 24 h, 48 h, and 72 h, respectively. The results showed that just a few contaminations were found after 72 h. However, no contamination was found when the porcine skin explant wells incubated with the PAO1 culture after 72 h, which implied the model was suitable for studying *P. aeruginosa* biofilm development in chronic wounds.

### Transcriptome analysis at genome-wide level

In order to identify genes that are specifically expressed in biofilms, RNA-seq technique was used to determine the transcriptome of *P. aeruginosa* PAO1 for mature biofilm and planktonic cells. The total number of mapped reads ranged between 35,183,947 and 37,580,850 for biofilm samples and between 36,481,656 and 39,427,607 for planktonic samples. 88.57% of total reads were successfully aligned to the reference genome, and 82.77% of all mapped reads were aligned to annotated gene regions for biofilm samples. For planktonic samples, 87.07% of mapped reads were aligned to annotated gene regions (Additional file 1: Table S1). In addition, blast alignment showed that the average rRNA contamination ratios were 4.88% and 3.62% for biofilm and planktonic samples, respectively (Additional file 1: Table S1), which indicated the data could be used for further analysis. Furthermore, RNA-seq data analysis showed that a total of 5,405 (97.00%) and 5,296 (95.05%) genes were confidently identified in biofilm and planktonic cells, respectively. Of which, 5,275 genes were both expressed in biofilm and planktonic cells. Above data analysis further determined that the *ex vivo* wound model was suitable for studying *P. aeruginosa* biofilms developed in chronic wounds without contaminations. Notably, in these expressed genes, 130 genes uniquely expressed in biofilm cells and 21 genes were found exclusively in planktonic cells. However, the expression levels of these genes that were only detected in either planktonic or biofilm cells were very low except that transcripts associated with the type II *hxc* secretion system expressed in biofilm state, but their transcription levels were not very high. (Additional file 1: Tables S2 and S3).

**Fig. 3:**
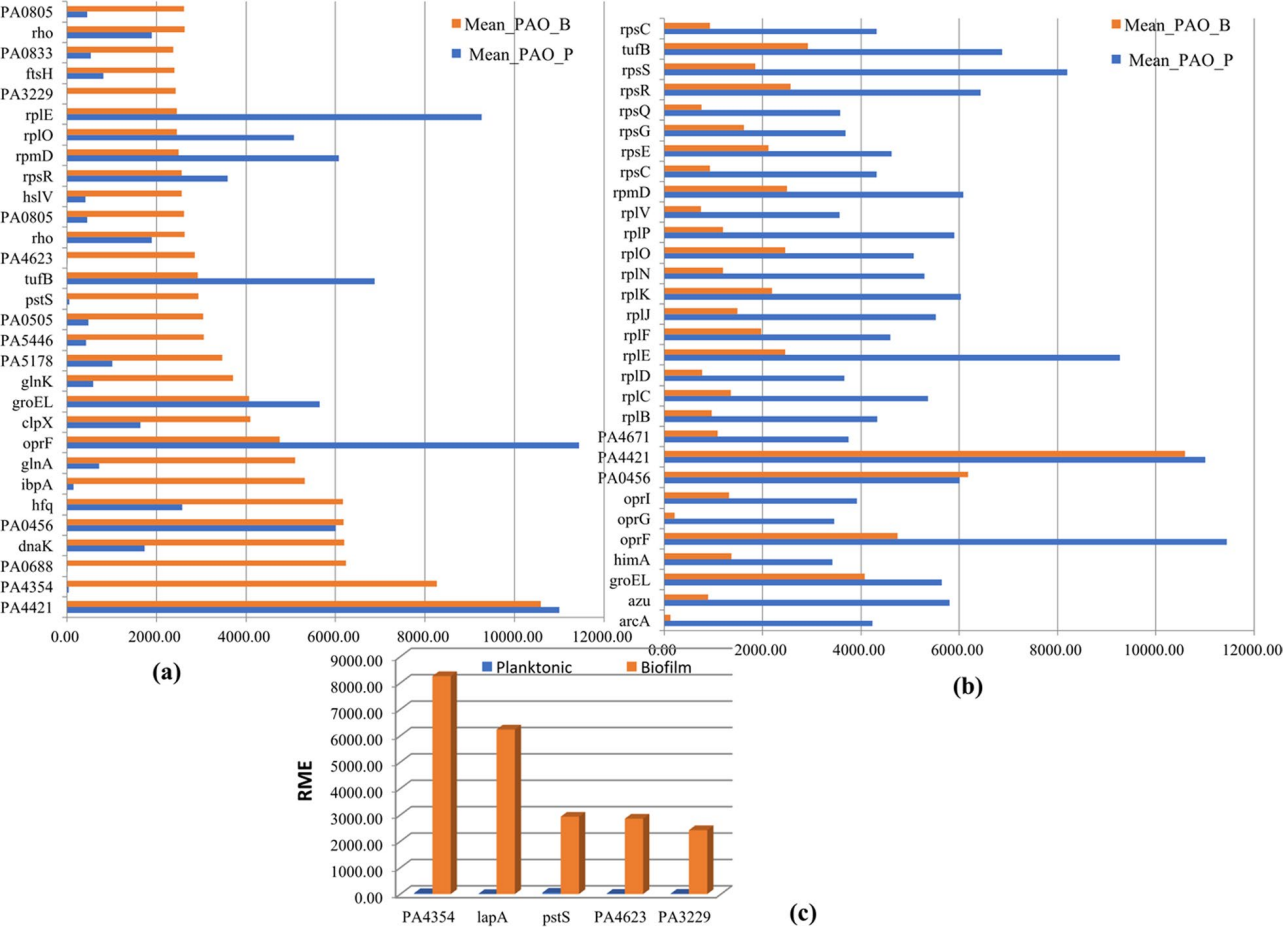
Relative mean expression (RME) levels of genes in biofilms and planktonic cells. RME values were calculated from the average values of normalized read counts in the biofilms (n = 3) and planktonic (n = 3) samples. **a** The 30 highest RME values were sorted in ascending order for genes in biofilm samples and are displayed with the RME values of corresponding genes in planktonic samples; **b** The 30 highest RME values were sorted in ascending order for genes in planktonic samples and are displayed with the RME values of corresponding genes in biofilm samples. **c** Genes highly expressed in biofilms but extremely low expression in planktonic cells

The combined expression levels of all genes were determined in biofilm and planktonic samples. The relative mean expression values (RME) for each identical gene product were produced and the top 30 most highly expressed genes in biofilms were ranked (Fig. 3a). The corresponding values in planktonic samples are also displayed together in Fig. 3a. Similarly, the top 30 most highly expressed genes in planktonic samples were ranked (Fig. 3b). In the biofilm cells, these genes, encoding alkaline phosphatase L, hypothetical protein, heat-shock protein LbpA, and glutamine synthetase, were among the most highly expressed genes with normalized matrix count value more than 5,000. Moreover, nine genes were enriched to biological process with cellular protein metabolic pathway (FDR < 0.001). However, among the 30 highest abundant genes in planktonic cells, 20 genes were enriched to translation process and ribosome pathway, which determined active metabolism in planktonic cells. A comparison of the 30 highest abundant genes in biofilm and planktonic cells resulted in an overlap of 9 genes. Of which, genes encoding cell division protein MraZ and ribosome were the most enriched. Significantly, the expression levels of 21 genes uniquely highly expressed in biofilm were very low in planktonic cells, especially the expression levels of *lap*A encoding alkaline phosphatase and *pst*S encoding phosphate ABC binding protein (Fig. 3c, Additional file 1: Table S4). In addition, 21 genes uniquely highly expressed in planktonic cells were related to ribosome. The expression levels of these genes were also high in biofilm cells, although they were not the top 30 highest expressed genes in biofilm cells. These differences suggested that there would be different functional features between biofilms and planktonic cells.

Gene Ontology (GO) analysis of the top 30 most abundant genes in biofilm and planktonic cells revealed that the transcripts associated with biological process of response to cellular protein metabolism were more enriched in planktonic cells than in biofilms (9 genes for biofilm, 22 genes for planktonic). Similarly, the Kyoto Encyclopedia of Genes and Genomes (KEGG) enrichment revealed that genes involved in ribosomal protein were more enriched in planktonic cells than biofilms (4 genes for biofilm, and 19 genes for planktonic).

### Enrichment analysis of differentially expressed genes

Sample to sample distance was calculated using R package DESeq2 (Love and Huber et al. 2014). The principal components analysis (PCA) showed that planktonic and biofilm samples formed distinct clusters (Additional file 1: Fig. S2). Meanwhile, the PCA plot displays larger differences among biofilm samples than planktonic samples, suggesting the functional features in the planktonic cells are more conserved than in the biofilm cells. In addition, the differentially expressed genes between biofilm and planktonic cells were also identified using R package DESeq2 Compared to planktonic cells, a total of 1205 (21.63%) genes were identified as differentially expressed in biofilms with 711 (12.76%) genes elevated. Among these genes, the most up-regulated genes were those associated with alkaline phosphatase, the type II *hxc* secretion system, phosphate ABC transporter, and others. Moreover, 62 of up-regulated genes were not expressed in planktonic samples, including genes associated with the type II *hxc* secretion system, assimilatory nitrate and nitrite reductases to ammonia, and hypothetical proteins. However, their expression levels in biofilms were not very high, ranged from 1.59 to 451.3 after normalization (Additional file 1: Table S5). Therefore, Therefore, these genes could not be suitable to differ biofilms from planktonic cells, but the genes controlled or regulated by these genes could be used as potential biomarker, for example, the expression levels of *lap*A controlled by the type II *hxc* secretion system was very high (6237 in biofilm but 2.6 in planktonic).In contrast, transcript levels of 494 (8.87%) genes were down-regulated in biofilms compared to planktonic cells, which involved in denitrification, pyochelin biosynthesis, the type VI secretion system, and others. Of which, 9 genes, related to the type III secretion system, cationic antimicrobial peptide (CAMP) resistance and pyochelin biosynthesis, were not expressed in biofilm samples, but their expression levels in planktonic samples were very low, except that the gene encoding unknown functional protein (Additional file 1: Table S6). Therefore, they cannot be used to differ biofilms from planktonic cells.

GO analysis and KEGG pathway enrichment of differentially expressed genes were performed using STRING (Szklarczyk et al. 2015). GO analysis showed that all of three processes were enriched among the genes with decreased transcription in biofilm cells. For biological process, 140 genes were enriched in biological process pathway, followed 132 genes in metabolic process pathway, 109 genes in cellular metabolic process pathway, and 107 genes in organic substance metabolic process pathway. For molecular function process, 136 genes were enriched in molecular function pathway, followed 101 genes in catalytic activity pathway. For cellular component process, 106 genes were enriched in cellular component pathway, and 104 genes in cell pathway. However, no process was enriched among the genes with increased transcription in biofilms, suggesting that biofilms were less metabolically active than planktonic cells. The KEGG pathways enrichment revealed that phosphate and phosphinate metabolism pathway and bacterial chemotaxis pathway were enriched among the transcripts that were elevated in biofilm cells; while two-component system pathway, ribosome, oxidative phosphorylation, and nitrogen metabolism were enriched among down-regulated genes. Interestingly, the processes and the pathways enriched among up-regulated genes were totally different from those among down-regulated genes, which indicate that there would be different metabolism between biofilm and planktonic cells.

Among up-regulated genes, three clusters were found, including one cluster related to the type II *hxc* secretion system comprising of 22 nodes with 53 edges, one associated with oxidative phosphorylation comprising of 13 nodes with 49 edges, and one involved in bacterial chemotaxis comprising of 8 nodes with 27 edges (Additional file 1: Fig. S3). Among down-regulated genes, three clusters were also found, including a cluster involved in denitrification comprising of 20 nodes with 70 edges, one involved in pyochelin biosynthesis comprising of 11 nodes with 24 edges, and one involved in the type VI secretion system comprising of 13 nodes with 35 edges (Additional file 1: Fig. S4).

### The top 20 most significantly up- and down-regulated genes in biofilms

The top 20 differentially expressed genes in both conditions, with the expression levels of these genes in either condition greater than 100 after normalization, are displayed in Table 1, and ranked according to the log_2_FC values. Interestingly, among the transcripts elevated in biofilms, 13 genes were clustered with medium confidence (0.40), including 8 genes involved in phosphonate and phosphinate metabolism pathway with highest confidence (0.90), alkaline phosphatase and pyrophosphate specific outer membrane porin OprO (Additional file 1: Fig. S5). Of which, *lap*A was the most up-regulated (log_2_FC = 11.32) with one of the top 30 most highly expressed genes. Another 7 genes without connected nodes were annotated hypothetical proteins.

**Table 1:** The top 20 up- and down-regulated genes in biofilm samples, respectively

Gene_id	Gene	Log_2_FC (PAO_B/PAO_P)	PAO_B	PAO_P	Gene description
PA0688	*lap*A	11.32	6237.32	2.60	Alkaline phosphatase L
PA3219	PA3219	9.85	395.65	0.46	Hypothetical protein
PA0842	PA0842	9.60	451.26	0.62	Glycosyl transferase family protein
PA3383	PA3383	9.18	767.95	1.42	Phosphonate ABC transporter substrate-binding protein
PA0691	PA0691	8.94	203.30	0.45	Hypothetical protein
PA4382	PA4382	8.72	442.90	1.13	Hypothetical protein
PA3382	*phn*E	8.71	267.00	0.69	Phosphonate transporter PhnE
PA4350	PA4350	8.65	1004.08	2.68	Hypothetical protein
PA3380	PA3380	8.29	243.00	0.83	Hypothetical protein
PA4623	PA4623	8.17	2857.47	10.66	Hypothetical protein
PA3378	PA3378	8.11	284.24	1.09	Hypothetical protein
PA3381	PA3381	8.09	203.83	0.80	Transcriptional regulator
PA3376	PA3376	8.01	188.62	0.79	Phosphonate C-P lyase system protein PhnK
PA3280	*opr*O	8.01	1907.03	7.95	Pyrophosphate-specific outer membrane porin OprO
PA3377	PA3377	8.00	374.09	1.57	Hypothetical protein
PA3384	*phn*C	7.99	406.79	1.71	Phosphonate ABC transporter ATP-binding protein
PA0692	PA0692	7.97	218.80	0.93	Hypothetical protein
PA4354	PA4354	7.96	8259.51	36.35	Hypothetical protein
PA0694	exbD2	7.96	260.44	1.14	Transporter ExbD
PA2803	PA2803	7.95	159.82	0.70	Hypothetical protein
PA1555.1	*cco*Q2	− 4.94	72.06	2569.51	Cytochrome C oxidase cbb3-type subunit CcoQ
PA5171	*arc*A	− 4.96	127.12	4229.36	Arginine deiminase
PA5373	*bet*B	− 4.99	27.43	927.48	Betaine aldehyde dehydrogenase
PA5172	*arc*B	− 5.00	94.84	3244.08	Ornithine carbamoyltransferase
PA0516	*nir*F	− 5.01	31.55	1090.83	Heme d1 biosynthesis protein NirF
PA4221	*fpt*A	− 5.10	4.54	165.71	Fe(III)-pyochelin outer membrane receptor
PA0518	*nir*M	− 5.11	39.30	1493.16	Cytochrome C-551
PA0049	PA0049	− 5.19	3.95	153.21	Hypothetical protein
PA5173	*arc*C	− 5.31	73.26	3107.41	Carbamate kinase
PA0519	*nir*S	− 5.41	60.36	2744.40	Nitrite reductase
PA2109	PA2109	− 5.86	1.75	107.96	Hypothetical protein
PA5372	*bet*A	− 5.99	3.10	209.46	Choline dehydrogenase
PA4225	*pch*F	-6.37	1.61	141.55	Pyochelin synthetase
PA4230	*pch*B	− 6.48	1.44	144.11	Isochorismate-pyruvate lyase
PA4888	*des*B	− 6.94	1.23	160.81	Acyl-CoA desaturase
PA2114	PA2114	− 7.02	4.57	631.58	Major facilitator superfamily transporter
PA2111	PA2111	− 7.26	5.09	835.52	Hypothetical protein
PA4889	PA4889	− 7.30	1.52	256.77	Oxidoreductase
PA2113	*opd*O	− 7.94	1.88	492.54	Pyroglutatmate porin OpdO
PA2112	PA2112	− 7.96	2.10	561.56	Hypothetical protein

Among the transcripts down-regulated in biofilm, the expression level of *nir*S gene, important for denitrification pathway, as well as the genes (*nir*F, *nir*M) with the functions of electron transport chain for denitrification pathway, was sharply decreased (log_2_FC < − 5.0). Therefore, NO, the product of nitrite reductase NirS, could be important to distinguish biofilms from planktonic cells. In addition, three genes associated with arginine deiminase pathway were also sharply down-regulated (log_2_FC about − 5.00) suggesting that biofilms had significantly decreased level of metabolism.

**Fig. 4:**
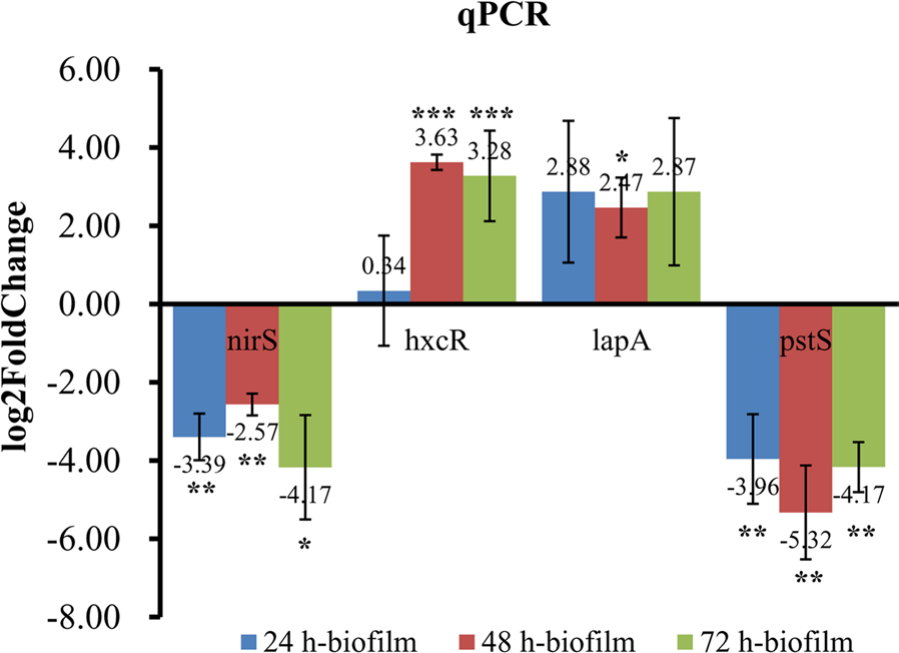
Quantification of transcription of genes involved in the novel type II *hxc* secretion system and denitrification pathway in biofilm cells for different time. The data indicate the log_2_FoldChange expression of genes in biofilm cells compared to planktonic cells. The bars represent the mean and the standard error of the mean (mean ± SD). *P* values that are significantly different by T.test are indicated by asterisks as follows: *, *P* < 0.05; **, *P* < 0.01; ***, *P* < 0.001

To identify our RNA-seq analysis, we verified parts of these up- and down-regulated genes using RT-qPCR experiment. Expression levels of these tested genes were consistent with the data obtained from RNA-seq experiment except the expression of *pst*S (Additional file 1: Fig. S6). RNA-seq data analysis revealed that no single gene was found as very high expression in one form of bacterial organization but completely absent in another one. However, in our data, the expression levels of those genes associated with the type II *hxc* secretion system, phosphate metabolism, and denitrification were significantly changed in biofilm state compared to planktonic state. Therefore, transcription levels of parts of these genes related to above functions were further confirmed with the RT-qPCR experiments on biofilms incubated in the porcine skin explant wells for 24 h, 48 h, and 72 h, respectively. The results demonstrated that expression levels of *lap*A were higher at the whole biofilm developing cycle than at planktonic state; while the transcription levels of *nir*S were decreased compared to planktonic state (Fig. 4). Incredibly, the expression of *pst*S was decreased in biofilms compared to planktonic state. Therefore, alkaline phosphatase LapA would serve as potential marker to monitor chronic wound infections by *P. aeruginosa* biofilms, and inducing NO or nitrite reductase would be used to inhibit chronic wound infections due to *P. aeruginosa* biofilms.

### Alkaline phosphatase is required for biofilm formation

**Fig. 5:**
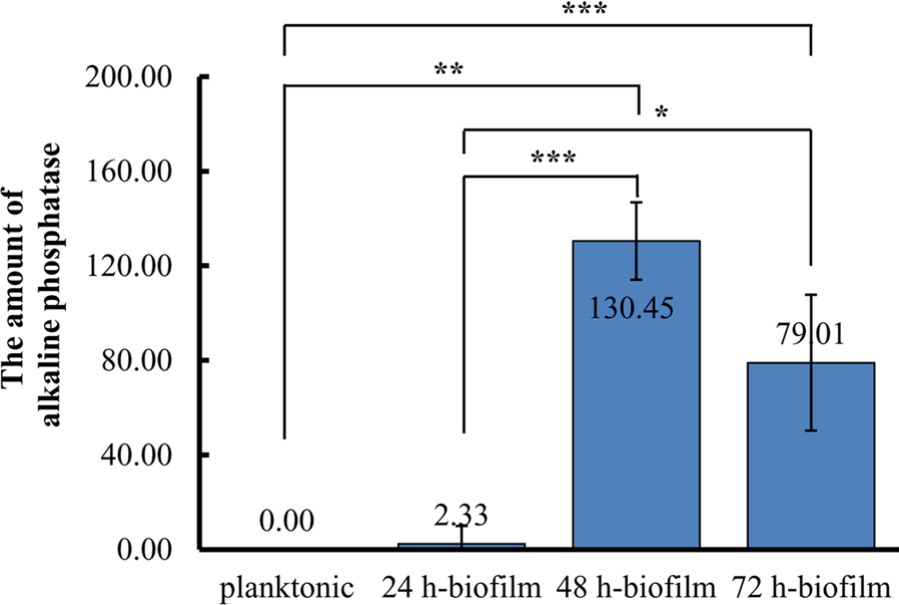
The amount of alkaline phosphatase measured in the supernatants from *P. aeruginosa* PAO1 at biofilm and planktonic states, respectively. The amount of alkaline phosphatase was defined as the µmoles of *p*-nitrophenol liberated from *p*-nitrophenyl phosphate at the specific time and the data was normalized to log_10_CFU. Data were collected from three independent experiments with at least three replicates each. Error bars represent the standard deviation from three independent experiments. *P* values that are significantly different by T.test are indicated by asterisks as follows: *, *P* < 0.05; **, *P* < 0.01; ***, *P* < 0.001

We also analyzed the extracellular alkaline phosphatase activity that was present in the supernatants of biofilms. Interestingly, the amount of secreted alkaline phosphatase was highest in mature biofilm, following in dispersal biofilms, and very less in early biofilms. However, PAO1 did not show significant alkaline phosphatase production in the supernatant at planktonic state (Fig. 5). In addition, alkaline phosphatase was not found in porcine skin explants as negative controls. Therefore, the results indicated that the release of alkaline phosphatase could contribute to *P. aeruginosa* biofilm formation in chronic wounds.

### Intracellular NO level is reduced in biofilms

**Fig. 6:**
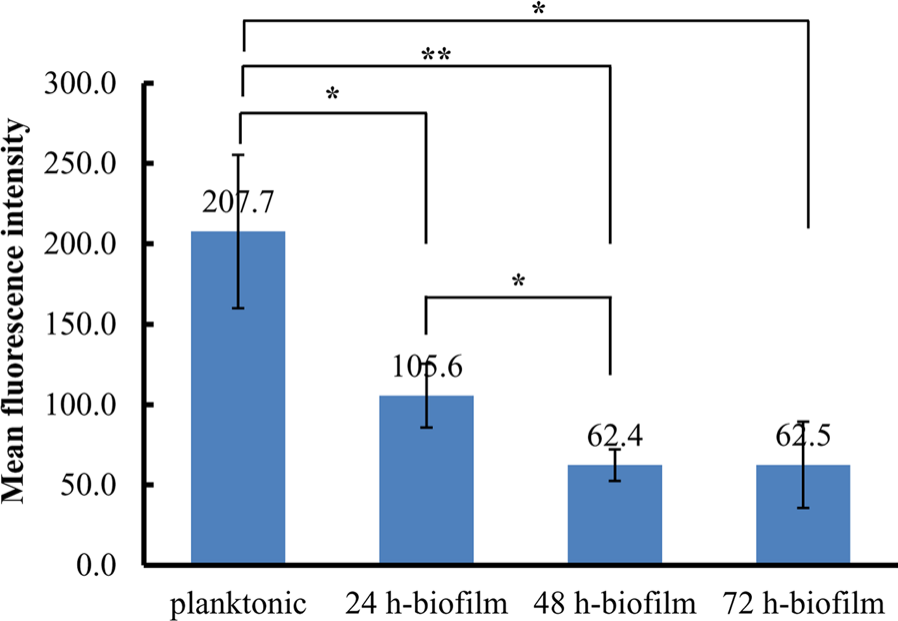
The mean fluorescence intensity of intracellular NO in *P. aeruginosa* PAO1 at biofilms and planktonic states, respectively. Data were collected from three independent experiments with at least three replicates each and normalized to log_10_CFU. Error bars represent the standard deviation from three independent experiments. *P* values that are significantly different by T.test are indicated by asterisks as follows: *, *P* < 0.05; **, *P* < 0.01; ***, *P* < 0.001

Both RNA-seq and RT-qPCR results showed that the transcription of *nir*S was decreased in biofilms compared to at planktonic state. Therefore, intracellular NO level was detected. The results showed that intracellular NO level was very high when PAO1 was at planktonic state. However, as biofilms growing, the intracellular NO level was going down. After biofilms were in maturation, the NO level was very low; while the NO level was a bit increased in dispersal biofilms (Fig. 6). Therefore, inducing intracellular NO production could be used to inhibit *P. aeruginosa* biofilms formation in chronic wounds.

## Discussion

In this work, the ex vivo chronic wound model with porcine skin explants were established followed our previously method with some modification (Yang et al. 2013). Although the tissue-based model employed here did not have any immune response to chronic wounds, the model could better mimic skin tissue than any other in vitro models, such as plastic microtiter plate, CDC, and flow-cell. While the biofilms developed on porcine skin may not identical to that found clinically, it presents a tradeoff necessary to achieve reproducible and controllable biofilms for study on wound infections due to biofilms (Wang et al. 2018).

Misic et al. (2014) found that among bacteria colonizing chronic wound tissue, *P. aeruginosa* was one of the predominant bacterium regardless of the etiology of the wound and the type of sampling (Tomic-Canic et al. 2020). The growth of *P. aeruginosa* in biofilm state renders such treatments infective, leading to chronic infections. Therefore, novel therapy strategies for chronic wound infections due to *P. aeruginosa* biofilms are urgently needed. As noted elsewhere, transcriptomics is the first stage of biofilm monitoring and studying tool, which can reveal important information about the adaption of a bacterial species to particular environmental niche through gene expression profiles, such as wounds (Wu et al. 2019; Seneviratne et al. 2020). In present study, we analyzed the transcriptome of chronic wound infections related *P. aeruginosa* cultivated under biofilm and planktonic conditions. The results demonstrated that more transcripts (711 genes) were increased in biofilms compared to planktonic cells. Importantly, our findings provide key insights into the development of biofilms and the pathogenicity of *P. aeruginosa* in chronic wound infections.

Here, we showed that wound-associated *P. aeruginosa* biofilm cells alter their gene expression profile, namely transcription levels of genes involved in translation (with down-regulation of genes encoding ribosomal proteins), the type II secretion system (with up-regulation of *hxc* locus and downstream gene *lap*A), type VI secretion system (with down-regulation of genes encoding H1-T6SS), and nitrogen metabolism, especially with down-regulation of genes associated denitrification. As a consequence, most biofilm cells are likely to encounter restricted availability of nutrients. Phosphate, an essential nutrient, has been recognized as an important signal that affects virulence in *P. aeruginosa* (Blus-Kadosh et al. 2013). *P. aeruginosa* has two phosphate uptake systems, a low-affinity (Pit); and a high affinity ABC transport (Pst) (Blus-Kadosh et al. 2013; Neznansky et al. 2014) showed that PstS not only mediated phosphate uptake from environment but also played an important role for biofilm formation. Meanwhile, they found that PstS secretion was dependent on the type II *hxc* secretion system, namely deletion of the *hxc*R gene almost entirely abolishes the presence of extracellular PstS (Neznansky et al. 2014). Therefore, the type II *hxc* secretion system could regulate *P. aeruginosa* biofilm formation through mediating PstS secretion. Interestingly, in our data, the expression of the whole *hxc* cluster was significantly up-regulated in biofilms compared to at planktonic state, which implied that the type II *hxc* secretion system would play an important role in *P. aeruginosa* biofilm formation in chronic wound infections. Moreover, Ball et al. (2012) determined that the *hxc* system in PAO1 was involved in secretion of a low molecular weight alkaline phosphatase encoded by the *lap*A gene, which was clustered to the type II *hxc* secretion locus. Their research further showed that the LapA may contribute *P. aeruginosa* to efficient colonization of the host through infecting *C. elegans* experiment (Ball et al. 2012). In addition, Neznansky (2014) also revealed that LapA was a central adhesion protein, which further determined that the type II *hxc* secretion system could be important for *P. aeruginosa* biofilm formation and virulence through elevating the expression of *pst*S and *lap*A. In our work, the transcriptomic data analysis showed that the expressions of *hxc*, *lap*A and *pst*S were up-regulated at biofilm state compare to planktonic state, especially *lap*A and *pst*S, which were among the top 30 most highly expression genes but very low expression in planktonic cells. However, the results of RT-qPCR assay showed that the expression levels of *lap*A and *hxc*R were increased at the whole biofilms developing cycle compared to at planktonic state. Surprisingly, the transcription of *pst*S was inconsistent with the result of RNA-seq analysis. Therefore, according to above analysis, the underlying mechanism of *P. aeruginosa* biofilm development in chronic wounds may be: wounds environment induced the expression of the type II *hxc* secretion system in *P. aeruginosa*, and then mediated the expression of *lap*A, which contributed *P. aeruginosa* to colonization in chronic wounds and biofilm formation. Subsequently, the results of our biochemical assay indicated that the alkaline phosphatase secreted in biofilms was more than at planktonic state. Therefore, *lap*A gene and its encoding protein could be used as potential biomarker to monitor *P. aeruginosa* biofilm in chronic wounds. Our coming work is to identify the hypothesis by means of *lap*A mutation and then animal model. Regarding of *pst*S, we are also going to investigate its function in chronic wound infections due to biofilms formation using wet experiments. Notably, the transcripts related to denitrification (NO_3_^−^→NO_2_^−^→NO→N_2_O→N_2_) were more abundant in planktonic cells, especially high expression of *nir*S gene encoding nitrite reductase to NO. Several studies have highlighted the role of NO in *P. aeruginosa* biofilm formation and dispersal (Cutruzzola and Frankenberg-Dinkel 2016; Park et al. 2020). In addition, Park’s research strongly suggested that nitrite transporter partially suppressed *nir*S expression in PAO1 so that *nir*S was expressed to specific levels to produce particular low level NO during biofilm formation, which might be a requirement for biofilm formation. Herein, we found that *nas*A encoding nitrite transporter was up-expressed but the expression of *nir*S was sharply down-regulated in biofilm cells, which is consistent with Park’s research. Moreover, the results of the intracellular NO detection assay showed that NO produced in *P. aeruginosa* at planktonic state was abundant compared to at the whole biofilms life cycle. Therefore, increasing NO or nitrite reductase NirS could be used to inhibit *P. aeruginosa* biofilms formation in chronic wounds. Our future work is to identify the hypothesis via overexpression of *nir*S gene and animal model.

Here, we observed that transcripts associated with the type VI secretion system (T6SS) were abundant in planktonic cells, which seemly conflicts with Tomic-Canic’s research (2020). The study of Tomic-Canic showed that the bacterium has an active T6SS in a chronic mode of infection by biofilm formation (Tomic-Canic et al. 2020). It is important to remember that our data represented a single snapshot of gene expression and had different model from Tomic-Canic’s, so even though these genes are relatively low in expression during chronic wound infections, they may still be important for bacterial fitness and could have been expressed at higher levels in earlier stage of infections. Furthermore, in our data, the transcripts associated with quorum sensing were not differentially expressed, which is inconsistent with previous researches on *P. aeruginosa* biofilm developed on the abiotic surfaces (Parsek and Greenberg 1999; Whiteley et al. 2001; Waite et al. 2005; Dotsch et al. 2012), but which is consistent with transcription levels of *P. aeruginosa* biofilm in humans infection (Cornforth et al. 2018). Therefore, the mechanism of biofilms developed in chronic wounds may be different from developed *in vitro*.

Taken together, these data indicated that *P. aeruginosa* changes its transcriptomic profile when growing as biofilms in chronic wounds. These changes are likely important for biofilm persistence and, consequently, for chronic wound infections by this bacterium. Furthermore, the fact that *lap*A controlled by the type II *hxc* secretion system was highly expressed in biofilms which represented an important finding. This might contribute toward a potential target for monitoring biofilms in chronic wounds by *P. aeruginosa*. In addition, the fact that the down-regulation of *nir*S in biofilms indicated that NO could be important to inhibit *P. aeruginosa* biofilm in chronic wounds.

## Supplementary Information

**Additional file 1.** Additional figures and tables.

## Data Availability

The RNA-Seq raw data had been deposited at the Sequence Read Archive (SRA) with accession number PRJNA688537.
