# Supplementary material for: Transcriptional analysis and target genes discovery of Pseudomonas aeruginosa biofilm developed ex vivo chronic wound model
Source: AMB Express. 2021 Nov 27;11:157. doi: 10.1186/s13568-021-01317-2 (PMC8627541; doi:10.1186/s13568-021-01317-2)
Supplement: Supplementary file 1 — Additional file 1. Additional figures and tables. [file 13568_2021_1317_MOESM1_ESM.pdf]

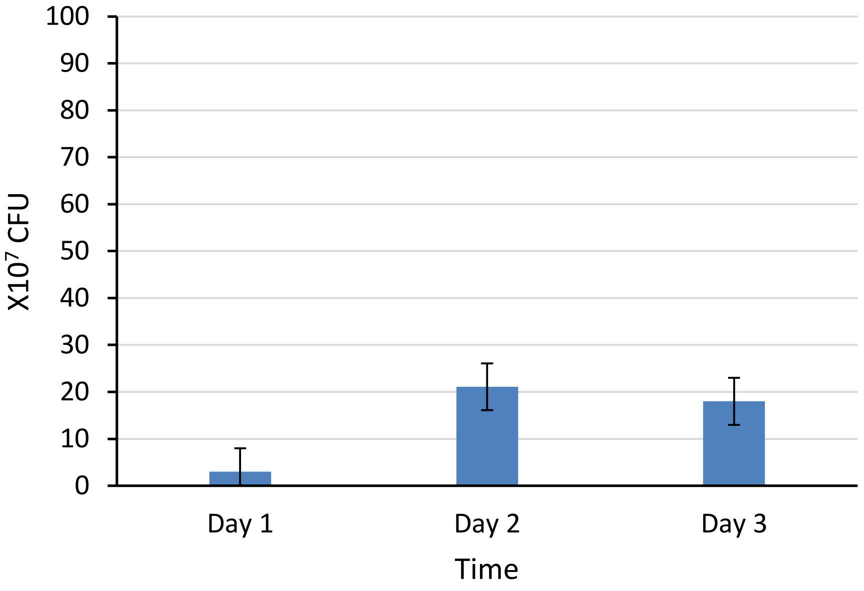

Fig. S1 Number of viable *P. aeruginosa* PAO1 (x 10^7 CFU/well) in porcine skin explant well.

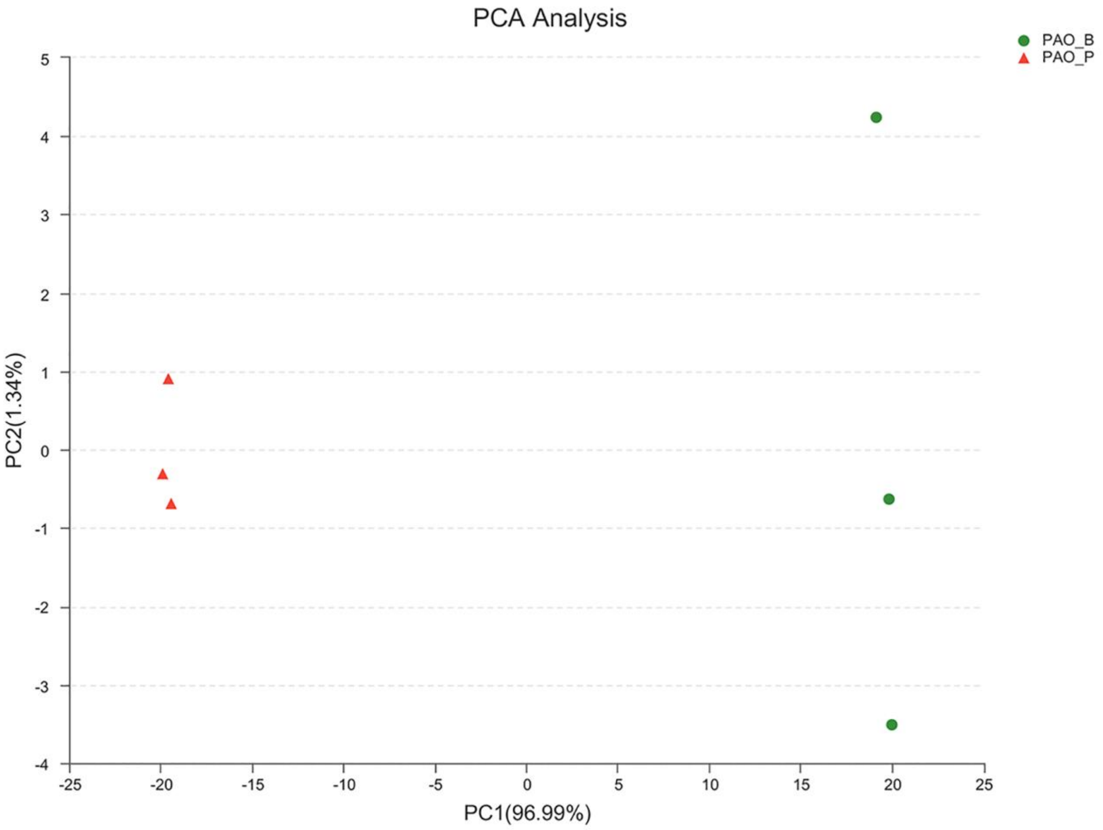

Fig. S2 Global gene expression between biofilm and planktonic cells. Principal component analysis (PCA) plot displaying sample-to-sample distance for planktonic and biofilm samples, which was carried out using the R package DESeq2.

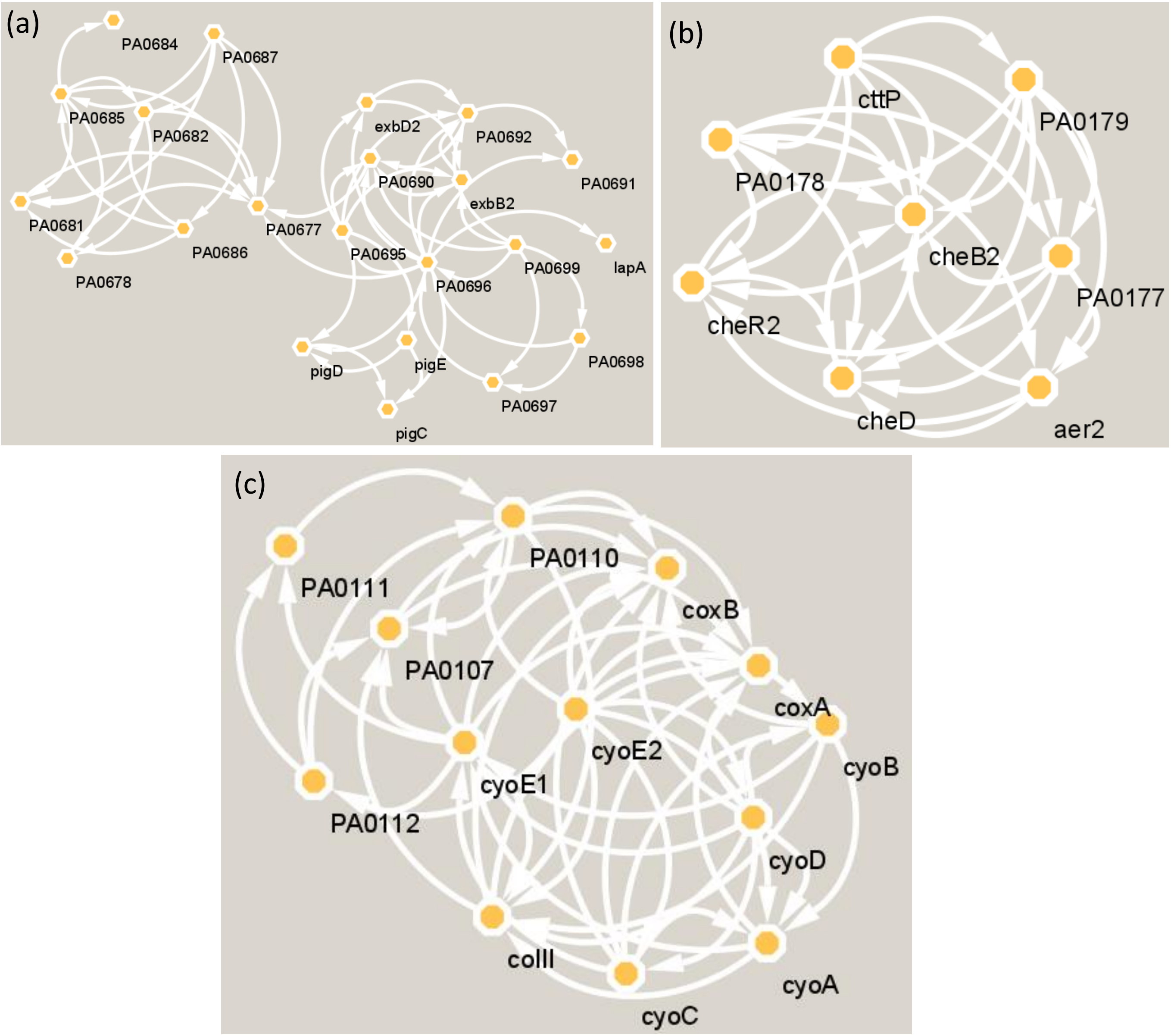

Fig. S3 Protein-protein interaction network construction and clusters identification for up-regulated genes in biof ilm cells, which was carried out using STRING observed with Cytoscape. (a), the cluster associated with the novel type II secretion system; (b), the cluster associated with bacterial chemotaxis; (c), the cluster associated with oxi dative phosphorylation.

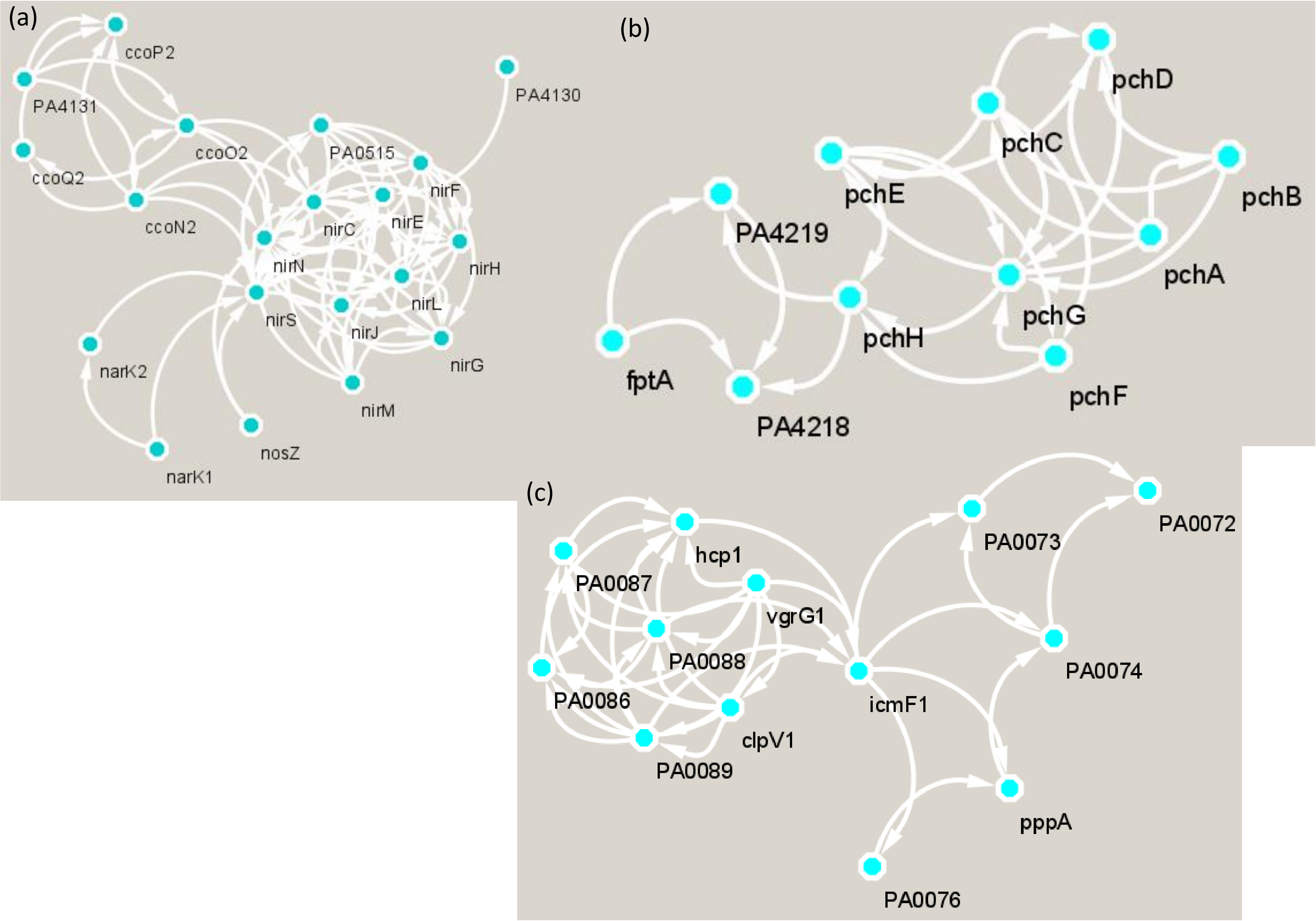

Fig. S4 Protein-protein interaction network construction and clusters identification for down-regulated genes in biofilm cells, which was carried out using STRING observed with Cytoscape. (a), the cluster involved in denitrification; (b), the cluster involved in pyochelin synthesis; (c), the cluster involved in the type VI secretion system.

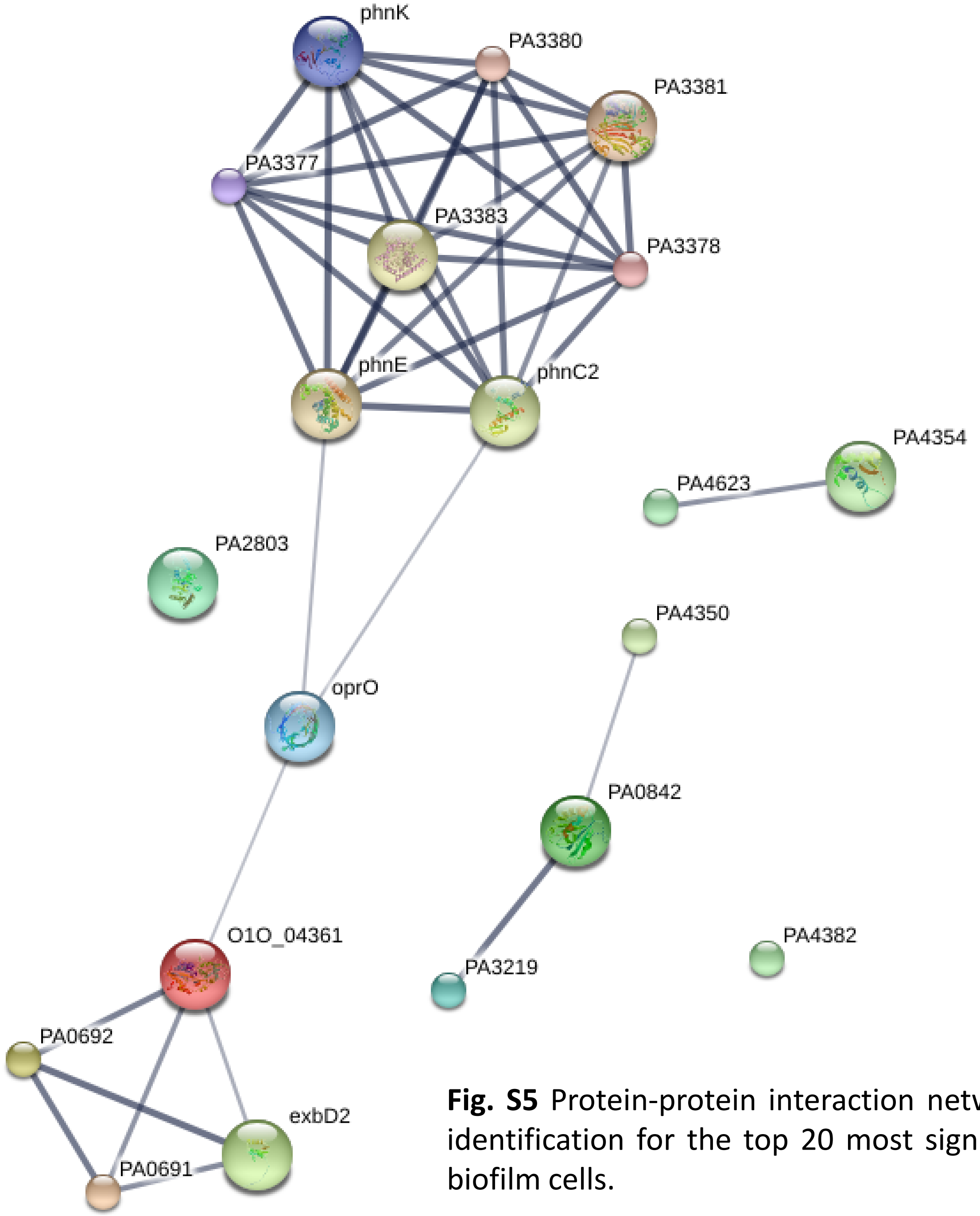

Fig. S5 Protein-protein interaction network construction and clusters identification for the top 20 most significantly up-regulated genes in biofilm cells.

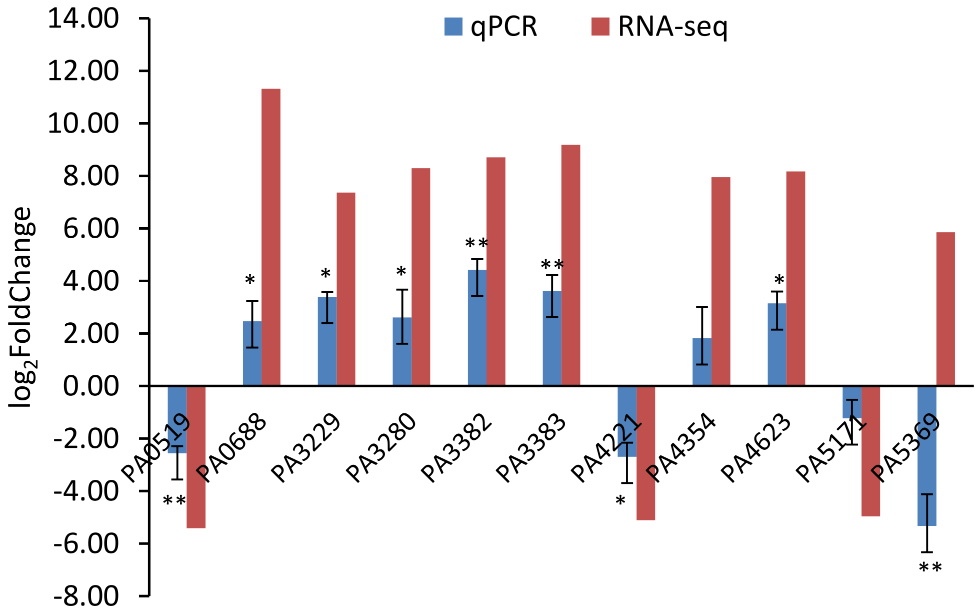

Fig. S6 RT-qPCR validation of the transcription of differentially expressed gene. Net total RNAs used were obtaine d from independent experiments performed under the same biological conditions as RNA-seq libraries constructi on (48 h). The data indicate the log_2FoldChange expression of genes in biofilm cells compared to planktonic cells. For qPCR experiments, the bars represent the mean and the standard error of the mean (Mean ± SD). **P* ≤ 0.05, ***P* ≤ 0.01, ****P* ≤ 0.001.

Table S1. RNA-seq read counts summary of biofilm and planktonic samples by *P. aeruginosa* PAO1

Sample Name	Total Reads	Genome Mapped Reads	Genome Mapped Ratio(%)	Unmapped Reads	Unmapped Reads Ratio(%)	Uniq Mapped Reads	Uniq Mapped Reads Ratio(%)	rRNA Ratio(%)
PAO_B1	39,525,628	35,183,947	89.02	4,341,681	10.98	32,658,653	82.63	5.429
PAO_B2	41,069,050	36,399,250	88.63	4,669,800	11.37	34,279,068	83.47	4.206
PAO_B3	42,674,218	37,580,850	88.06	5,093,368	11.94	35,084,045	82.21	4.996
PAO_P1	39,423,340	36,481,656	92.54	2,941,684	7.46	34,197,230	86.74	3.991
PAO_P2	42,617,410	39,427,607	92.52	3,189,803	7.48	37,374,307	87.7	2.956
PAO_P3	40,534,714	37,492,739	92.5	3,041,975	7.5	35,171,635	86.77	3.921

Table S2. Genes uniquely expressed in the biofilm cells.

Gene_id	Gene_name	Mean_PAO_B	Gene_description
PA0136	PA0136	10.51	ABC transporter ATP-binding protein
PA0189	PA0189	1.01	porin
PA0241	PA0241	1.23	major facilitator superfamily transporter
PA0273	PA0273	1.08	major facilitator superfamily transporter
PA0677	PA0677	42.67	HxcW pseudopilin
PA0678	PA0678	88.84	HxcU pseudopilin
PA0680	PA0680	70.59	HxcV pseudopilin
PA0683	PA0683	26.25	type II secretion system protein
PA0686	PA0686	49.38	type II secretion system protein HxcR
PA0687	PA0687	43.52	type II secretion system protein
PA0689	PA0689	2.41	hypothetical protein
PA0691	PA0691	203.30	hypothetical protein
PA0692	PA0692	218.80	hypothetical protein
PA0693	exbB2	206.47	transporter ExbB
PA0786	PA0786	1.37	transporter
PA0842	PA0842	451.26	glycosyl transferase family protein
PA0885	PA0885	1.61	C4-dicarboxylate transporter
PA0996	pqsA	2.31	anthranilate--CoA ligase
PA1108	PA1108	2.39	major facilitator superfamily transporter
PA1130	rhlC	1.77	rhamnosyltransferase
PA1146	PA1146	1.28	iron-containing alcohol dehydrogenase
PA1147	PA1147	1.59	amino acid permease
PA1168	PA1168	3.58	hypothetical protein
PA1186	PA1186	1.31	hypothetical protein
PA1187	PA1187	7.39	acyl-CoA dehydrogenase
PA1212	PA1212	2.28	major facilitator superfamily transporter
PA1214	PA1214	1.44	hypothetical protein
PA1217	PA1217	1.26	2-isopropylmalate synthase
PA1218	PA1218	1.18	hypothetical protein
PA1232	PA1232	1.36	hypothetical protein
PA1233	PA1233	2.28	hypothetical protein
PA1238	PA1238	1.91	multidrug efflux pump outer membrane protein
PA1248	aprF	5.76	alkaline protease secretion protein AprF
PA1251	PA1251	1.67	chemotaxis transducer

PA1253	PA1253	3.74	semialdehyde dehydrogenase
PA1265	PA1265	1.43	hypothetical protein
PA1266	PA1266	3.08	oxidoreductase
PA1356	PA1356	11.14	hypothetical protein
PA1412	PA1412	1.33	hypothetical protein
PA1419	PA1419	21.66	transporter
PA1568	PA1568	5.24	hypothetical protein
PA1569	PA1569	7.70	major facilitator superfamily transporter
PA1779	PA1779	23.97	assimilatory nitrate reductase
PA1780	nirD	40.54	assimilatory nitrite reductase small subunit
PA1781	nirB	71.07	assimilatory nitrite reductase large subunit
PA1782	PA1782	9.17	serine/threonine-protein kinase
PA1783	nasA	25.62	nitrate transporter
PA1785	PA1785	19.12	hypothetical protein
PA1849	PA1849	2.15	hypothetical protein
PA1870	PA1870	2.46	hypothetical protein
PA1896	PA1896	1.22	hypothetical protein
PA1897	PA1897	1.95	hypothetical protein
PA1923	PA1923	3.29	cobaltochelatase subunit CobN
PA1924	PA1924	2.30	hypothetical protein
PA1925	PA1925	6.21	hypothetical protein
PA1972	PA1972	70.74	hypothetical protein
PA2055	PA2055	2.86	major facilitator superfamily transporter
PA2058	PA2058	2.26	ABC transporter
PA2059	PA2059	2.51	ABC transporter permease
PA2060	PA2060	2.94	ABC transporter permease
PA2064	pcoB	1.26	copper resistance protein B
PA2065	pcoA	2.15	copper resistance protein A
PA2068	PA2068	1.51	major facilitator superfamily transporter
PA2077	PA2077	128.49	hypothetical protein
PA2135	PA2135	1.10	transporter
PA2136	PA2136	2.26	hypothetical protein
PA2146	PA2146	2.63	hypothetical protein
PA2154	PA2154	1.45	hypothetical protein
PA2178	PA2178	2.77	hypothetical protein
PA2181	PA2181	1.79	glutamate--cysteine ligase
PA2213	PA2213	1.41	porin

PA2214	PA2214	1.22	major facilitator superfamily transporter
PA2304	ambC	1.19	protein AmbC
PA2314	PA2314	1.50	major facilitator superfamily transporter
PA2335	PA2335	2.29	TonB-dependent receptor
PA2343	mtlY	1.07	xylulose kinase
PA2346	PA2346	3.64	hypothetical protein
PA2347	PA2347	1.93	hypothetical protein
PA2351	PA2351	4.21	ABC transporter permease
PA2416	treA	3.27	trehalase
PA2426	pvdS	63.66	extracytoplasmic-function sigma-70 factor
PA2465	PA2465	2.03	hypothetical protein
PA2472	PA2472	1.43	major facilitator superfamily transporter
PA2473	PA2473	1.04	glutathione S-transferase
PA2474	PA2474	1.25	hypothetical protein
PA2515	xylL	4.34	1%2C6-dihydroxycyclohexa-2%2C4-diene-1-carboxylate dehydrogenase
PA2520	czcA	1.58	resistance-nodulation-cell division (RND) divalent metal cation efflux transporter CzcA
PA2636	PA2636	36.46	hypothetical protein
PA2674	PA2674	1.17	type II secretion system protein
PA2688	pfeA	2.53	ferric enterobactin receptor
PA2689	PA2689	4.01	hypothetical protein
PA2701	PA2701	10.91	major facilitator superfamily transporter
PA2783	PA2783	1.09	hypothetical protein
PA2803	PA2803	159.82	hypothetical protein
PA2878	PA2878	4.34	hypothetical protein
PA2919	PA2919	17.13	hypothetical protein
PA2922	PA2922	1.25	hydrolase
PA2923	hisJ	1.62	histidine ABC transporter substrate-binding protein HisJ
PA2924	hisQ	1.61	histidine ABC transporter permease HisQ
PA2935	PA2935	2.25	hypothetical protein
PA2936	PA2936	15.05	hypothetical protein
PA3219	PA3219	395.65	hypothetical protein
PA3237	PA3237	3.53	hypothetical protein
PA3327	PA3327	1.25	non-ribosomal peptide synthetase
PA3334	PA3334	1.99	acyl carrier protein
PA3376	PA3376	188.62	phosphonate C-P lyase system protein PhnK
PA3380	PA3380	243.00	hypothetical protein
PA3381	PA3381	203.83	transcriptional regulator

PA3382	phnE	267.00	phosphonate transporter PhnE
PA3405	hasE	1.29	metalloprotease secretion protein
PA3518	PA3518	6.67	hypothetical protein
PA3519	PA3519	12.62	hypothetical protein
PA3521	PA3521	3.25	hypothetical protein
PA3541	alg8	3.03	glycosyltransferase alg8
PA3543	algK	2.01	alginate biosynthesis protein AlgK
PA3577	PA3577	7.92	hypothetical protein
PA3589	PA3589	1.08	acetyl-CoA acetyltransferase
PA4084	cupB3	4.35	usher CupB3
PA4087	PA4087	2.14	hypothetical protein
PA4096	PA4096	1.02	major facilitator superfamily transporter
PA4098	PA4098	1.11	short-chain dehydrogenase
PA4158	fepC	1.46	ferric enterobactin transporter FepC
PA4210	phzA1	1.02	phenazine biosynthesis protein
PA4814	fadH2	2.90	2%2C4-dienoyl-CoA reductase
PA4818	PA4818	1.05	hypothetical protein
PA4828	PA4828	7.17	hypothetical protein
PA4895	PA4895	2.67	transmembrane sensor
PA4903	PA4903	1.30	major facilitator superfamily transporter
PA5539	PA5539	6.36	GTP cyclohydrolase
PA5541	pyrQ	4.03	dihydroorotase

Table S3. Genes uniquely expressed in the planktonic cells.

Gene_id	Gene_name	Mean_PAO_P	Gene_description
PA0474	PA0474	1.43	esterase
PA1270	PA1270	1.49	hypothetical protein
PA1693	pscR	4.54	type III secretion system protein
PA1694	pscQ	2.91	type III secretion system protein
PA1695	pscP	1.57	translocation protein in type III secretion
PA1697	PA1697	1.79	type III secretion system ATPase
PA1702	PA1702	1.26	hypothetical protein
PA2110	PA2110	207.82	hypothetical protein
PA2208	PA2208	1.08	hypothetical protein
PA2286	PA2286	1.71	hypothetical protein
PA2514	antC	1.47	anthranilate dioxygenase reductase
PA3555	arnD	17.83	4-deoxy-4-formamido-L-arabinose-phosphoundecaprenol deformylase ArnD
PA3558	arnF	25.38	4-amino-4-deoxy-L-arabinose-phosphoundecaprenol flippase subunit ArnF
PA3591	PA3591	1.06	enoyl-CoA hydratase
PA3906	PA3906	3.27	hypothetical protein
PA3907	PA3907	2.05	hypothetical protein
PA4211	phzB1	1.11	phenazine biosynthesis protein
PA4222	PA4222	49.41	ABC transporter ATP-binding protein
PA4229	pchC	49.68	pyochelin biosynthetic protein PchC
PA4652	PA4652	1.20	hypothetical protein
PA4823	PA4823	1.02	hypothetical protein

Table S4. The 30 highest expressed genes in biofilm and planktonic cells, respectively.

Gene_id	Gene_name	Mean_PAO_B	Mean_PAO_P	Gene_description
**Biofilm**				
PA4421	PA4421	10592.19	10999.75	cell division protein MraZ
PA4354	PA4354	8259.51	36.35	hypothetical protein
PA0688	PA0688	6237.32	2.60	alkaline phosphatase L
PA4761	dnaK	6193.85	1731.47	molecular chaperone DnaK
PA0456	PA0456	6184.67	6007.37	cold-shock protein
PA4944	hfq	6166.50	2578.56	RNA-binding protein Hfq
PA3126	ibpA	5311.63	141.26	heat-shock protein IbpA
PA5119	glnA	5105.36	715.58	glutamine synthetase
PA1777	oprF	4745.76	11443.85	outer membrane porin F
PA1802	clpX	4091.78	1636.08	ATP-dependent protease ATP-binding subunit ClpX
PA4385	groEL	4075.18	5641.20	molecular chaperone GroEL
PA5288	glnK	3705.19	586.88	nitrogen regulatory protein P-II 2
PA5178	PA5178	3473.40	1016.85	hypothetical protein
PA5446	PA5446	3056.05	426.05	hypothetical protein
PA0505	PA0505	3043.43	479.53	hypothetical protein
PA5369	pstS	2932.52	54.24	phosphate ABC transporter substrate-binding protein
PA4277	tufB	2917.49	6871.17	elongation factor Tu
PA4623	PA4623	2857.47	10.66	hypothetical protein
PA5239	rho	2624.37	1894.40	transcription termination factor Rho
PA0805	PA0805	2613.78	457.37	hypothetical protein
PA5053	hslV	2567.38	418.38	ATP-dependent protease peptidase subunit
PA4934	rpsR	2566.24	3584.29	30S ribosomal protein S18
PA4245	rpmD	2499.43	6078.65	50S ribosomal protein L30
PA4244	rplO	2461.69	5076.87	50S ribosomal protein L15
PA4251	rplE	2456.29	9264.08	50S ribosomal protein L5
PA3229	PA3229	2422.88	15.59	hypothetical protein
PA4751	ftsH	2400.59	813.93	cell division protein FtsH
PA0833	PA0833	2370.85	527.13	hypothetical protein
PA5239	rho	2624.37	1894.40	transcription termination factor Rho
PA0805	PA0805	2613.78	457.37	hypothetical protein
**Planktonic**				
PA5171	arcA	4229.36	127.12	arginine deiminase
PA4922	azu	5797.36	893.43	azurin
PA4385	groEL	5641.20	4075.18	molecular chaperone GroEL

PA2738	himA	3424.88	1360.14	integration host factor subunit alpha
PA1777	oprF	11443.85	4745.76	outer membrane porin F
PA4067	oprG	3461.93	208.83	outer membrane protein OprG
PA2853	oprI	3913.19	1317.51	outer membrane lipoprotein OprI
PA0456	PA0456	6007.37	6184.67	cold-shock protein
PA4421	PA4421	10999.75	10592.19	cell division protein MraZ
PA4671	PA4671	3743.77	1089.02	50S ribosomal protein L25/general stress protein Ctc
PA4260	rplB	4330.63	969.11	50S ribosomal protein L2
PA4263	rplC	5367.86	1350.29	50S ribosomal protein L3
PA4262	rplD	3658.94	775.75	50S ribosomal protein L4
PA4251	rplE	9264.08	2456.29	50S ribosomal protein L5
PA4248	rplF	4599.86	1970.62	50S ribosomal protein L6
PA4272	rplJ	5517.72	1490.18	50S ribosomal protein L10
PA4274	rplK	6032.37	2189.69	50S ribosomal protein L11
PA4253	rplN	5296.34	1198.01	50S ribosomal protein L14
PA4244	rplO	5076.87	2461.69	50S ribosomal protein L15
PA4256	rplP	5902.73	1195.75	50S ribosomal protein L16
PA4258	rplV	3570.49	742.85	50S ribosomal protein L22
PA4245	rpmD	6078.65	2499.43	50S ribosomal protein L30
PA4257	rpsC	4314.83	930.11	30S ribosomal protein S3
PA4246	rpsE	4629.42	2114.69	30S ribosomal protein S5
PA4267	rpsG	3686.27	1625.19	30S ribosomal protein S7
PA4254	rpsQ	3584.29	755.36	30S ribosomal protein S17
PA4934	rpsR	6435.96	2566.24	30S ribosomal protein S18
PA4259	rpsS	8203.24	1846.34	30S ribosomal protein S19
PA4277	tufB	6871.17	2917.49	elongation factor Tu
PA4257	rpsC	4314.83	930.11	30S ribosomal protein S3

Table S5. The genes of elevated expression in biofilm not expressed in planktonic cells.

Gene_id	Gene name	Log2FC(PAO_B/PAO_P)	PAO_B	PAO_P	Gene description
PA0842	PA0842	9.60	451.26	0.62	glycosyl transferase family protein
PA3219	PA3219	9.85	395.65	0.46	hypothetical protein
PA3382	phnE	8.71	267.00	0.69	phosphonate transporter PhnE
PA3380	PA3380	8.29	243.00	0.83	hypothetical protein
PA0692	PA0692	7.97	218.80	0.93	hypothetical protein
PA0693	exbB2	7.84	206.47	0.96	transporter ExbB
PA3381	PA3381	8.09	203.83	0.80	transcriptional regulator
PA0691	PA0691	8.94	203.30	0.45	hypothetical protein
PA3376	PA3376	8.01	188.62	0.79	phosphonate C-P lyase system protein PhnK
PA2803	PA2803	7.95	159.82	0.70	hypothetical protein
PA2077	PA2077	7.24	128.49	0.90	hypothetical protein
PA0678	PA0678	7.79	88.84	0.44	HxcU pseudopilin
PA1781	nirB	7.64	71.07	0.38	assimilatory nitrite reductase large subunit
PA1972	PA1972	6.36	70.74	0.93	hypothetical protein
PA0680	PA0680	8.00	70.59	0.30	HxcV pseudopilin
PA2426	pvdS	6.35	63.66	0.84	extracytoplasmic-function sigma-70 factor
PA0686	PA0686	6.42	49.38	0.62	type II secretion system protein HxcR
PA0687	PA0687	6.15	43.52	0.66	type II secretion system protein
PA0677	PA0677	7.34	42.67	0.28	HxcW pseudopilin
PA1780	nirD	7.80	40.54	0.20	assimilatory nitrite reductase small subunit
PA2636	PA2636	5.45	36.46	0.90	hypothetical protein
PA0683	PA0683	5.23	26.25	0.75	type II secretion system protein
PA1783	nasA	6.01	25.62	0.43	nitrate transporter
PA1779	PA1779	5.20	23.97	0.70	assimilatory nitrate reductase
PA1785	PA1785	4.53	19.12	0.90	hypothetical protein
PA2919	PA2919	5.75	17.13	0.36	hypothetical protein
PA2936	PA2936	4.41	15.05	0.76	hypothetical protein
PA3519	PA3519	4.14	12.62	0.76	hypothetical protein
PA1356	PA1356	5.18	11.14	0.33	hypothetical protein
PA2701	PA2701	3.74	10.91	0.87	major facilitator superfamily transporter
PA0136	PA0136	3.64	10.51	0.91	ABC transporter ATP-binding protein
PA1782	PA1782	4.22	9.17	0.53	serine/threonine-protein kinase
PA1569	PA1569	3.15	7.70	0.92	major facilitator superfamily transporter
PA1187	PA1187	3.20	7.39	0.86	acyl-CoA dehydrogenase

PA4828	PA4828	3.11	7.17	0.89	hypothetical protein
PA3518	PA3518	3.68	6.67	0.56	hypothetical protein
PA5539	PA5539	3.18	6.36	0.74	GTP cyclohydrolase
PA1925	PA1925	3.66	6.21	0.54	hypothetical protein
PA1248	aprF	2.76	5.76	0.91	alkaline protease secretion protein AprF
PA1568	PA1568	2.95	5.24	0.74	hypothetical protein
PA4084	cupB3	2.78	4.35	0.67	usher CupB3
PA2515	xylL	2.95	4.34	0.60	1%2C6-dihydroxycyclohexa-2%2C4-diene-1-carboxylate dehydrogenase
PA2878	PA2878	2.56	4.34	0.79	hypothetical protein
PA2351	PA2351	2.41	4.21	0.86	ABC transporter permease
PA5541	pyrQ	2.73	4.03	0.64	dihydroorotase
PA2689	PA2689	2.85	4.01	0.59	hypothetical protein
PA1253	PA1253	2.16	3.74	0.89	semialdehyde dehydrogenase
PA2346	PA2346	2.29	3.64	0.79	hypothetical protein
PA1168	PA1168	2.25	3.58	0.83	hypothetical protein
PA2416	treA	2.13	3.27	0.79	trehalase
PA3541	alg8	2.21	3.03	0.70	glycosyltransferase alg8
PA4814	fadH2	2.32	2.90	0.63	2%2C4-dienoyl-CoA reductase
PA2055	PA2055	2.12	2.86	0.70	major facilitator superfamily transporter
PA2178	PA2178	2.20	2.77	0.66	hypothetical protein
PA2059	PA2059	2.05	2.51	0.64	ABC transporter permease
PA0689	PA0689	8.23	2.41	0.00	hypothetical protein
PA1108	PA1108	2.26	2.39	0.53	major facilitator superfamily transporter
PA1924	PA1924	2.29	2.30	0.50	hypothetical protein
PA2136	PA2136	2.52	2.26	0.43	hypothetical protein
PA1238	PA1238	2.20	1.91	0.44	multidrug efflux pump outer membrane protein
PA2181	PA2181	2.12	1.79	0.44	glutamate--cysteine ligase
PA1147	PA1147	2.36	1.59	0.33	amino acid permease
PA3406	hasD	2.64	0.83	0.14	transporter HasD
PA2522	czcC	3.18	0.78	0.09	outer membrane protein CzcC

Table S6. The genes of elevated expression in planktonic cells not expressed in biofilm.

Gene_id	Gene name	Log2FC(PAO_B/PAO_P)	PAO_B	PAO_P	Gene description
PA2110	PA2110	-8.11	0.71	207.82	hypothetical protein
PA4229	pchC	-6.49	0.52	49.68	pyochelin biosynthetic protein PchC
PA4222	PA4222	-5.60	0.95	49.41	ABC transporter ATP-binding protein
PA3558	arnF	-4.76	0.86	25.38	4-amino-4-deoxy-L-arabinose-phosphoundecaprenol flippase subunit ArnF
PA3555	arnD	-4.19	0.92	17.83	4-deoxy-4-formamido-L-arabinose-phosphoundecaprenol deformylase ArnD
PA1693	pscR	-2.36	0.82	4.54	type III secretion system protein
PA3906	PA3906	-2.92	0.40	3.27	hypothetical protein
PA1694	pscQ	-3.34	0.27	2.91	type III secretion system protein
PA1695	pscP	-2.43	0.27	1.57	translocation protein in type III secretion

Table S7. Primers used in RT-qPCR experiments.

Gene name	Annotation	Primer sequence (5' to 3')	Amplicon size (bp)
PA0519	nitrite reductase	CTGGAACCGAAGCAGATCGT<br>TTGACGATGAACTCGGGGTG	119
PA0688	alkaline phosphatase L	GTTCACTATGCCGGTAGCGA<br>CCGTAGGTGCCGTTGTAGTT	80
PA3229	hypothetical protein	ACCGTACCGACAATTCGACC<br>CCATTCGAGGAAGCGGATCT	98
PA3280	pyrophosphate-specific outer membrane porin OprO	TACAGCCGTTTCGACGGTTT<br>TCAGGTTGACCGGGTTGAAG	211
PA3382	phosphonate transporter PhnE	AGAACATCGTGCCCTGGTG<br>ATGGCGAAGACCATCTCGTT	85
PA3383	phosphonate ABC transporter substrate-binding protein	AAACGCTTCAGTCGTGTGCT<br>TTGAGGTTCTGCGACGACTC	125
PA4221	Fe(III)-pyochelin outer membrane receptor	AGGCAAGACCTACGAAACCG<br>TGCGGATTGTTCTCCAGGTC	99
PA4354	hypothetical protein	TCTCTCCCAGTCCACCGTAT<br>CTCCTCGTTGCGCTTGAAGA	106
PA4623	hypothetical protein	CAACAACTGATCGCCTCCGA<br>AAGCGATCGATGGTCTGCTC	125
PA5171	arginine deiminase	GAGCAATGGGACGACGGTAA<br>GTGTTGGTGTAGGTGTTGCG	83
PA5369	phosphate ABC transporter substrate-binding protein	GTACGGCTATAAGCCGACCG<br>GAAGATCGCATCGACCTGCT	112
PA0393(proC)	pyrroline-5-carboxylate reductase	ATCGACGTGGTCGAGTCCAA<br>TCAGTTGCTCCGGCTTCAG	127
PA3617(recA)	recombinase A	GCCAACTGCCTGGTCATCTT<br>GGCGTAGAACTTCAGTGCGT	105
